# Health Effects of Fructose and Fructose-Containing Caloric Sweeteners: Where Do We Stand 10 Years After the Initial Whistle Blowings?

**DOI:** 10.1007/s11892-015-0627-0

**Published:** 2015-06-24

**Authors:** Luc Tappy, Kim-Anne Lê

**Affiliations:** Department of Physiology, Faculty of Biology and Medicine, University of Lausanne, Rue du Bugnon 7, 1005 Lausanne, Switzerland; Department of Public Health Nutrition, Nestlé Research Center, Vers-chez-les-blanc, Route du Jorat 57, 1000 Lausanne 26, Switzerland

**Keywords:** Sugars, Fructose, Metabolic syndrome, Insulin resistance, Lipotoxicity, Cardiovascular diseases

## Abstract

Suspicion that fructose-containing caloric sweeteners (FCCS) may play a causal role in the development of metabolic diseases has elicited intense basic and clinical research over the past 10 years. Prospective cohort studies converge to indicate that FCCS, and more specifically sugar-sweetened beverages (SSBs), consumption is associated with weight gain over time. Intervention studies in which FCCS or SSB consumption is altered while food intake is otherwise left ad libitum indicate that increased FCCS generally increases total energy intake and body weight, while FCCS reduction decreases body weight gain. Clinical trials assessing the effects of SSB reduction as a sole intervention however fail to observe clinically significant weight loss. Many mechanistic studies indicate that excess FCCS can cause potential adverse metabolic effects. Whether this is associated with a long-term risk remains unknown. Scientific evidence that excess FCCS intake causes more deleterious effects to health than excess of other macronutrients is presently lacking. However, the large consumption of FCCS in the population makes it one out of several targets for the treatment and prevention of metabolic diseases.

## Introduction

Free fructose and sucrose are naturally present in fruits, vegetables, and honey, and have most likely been part of the human diet since the beginning of mankind. The level of fructose consumption remained however very low until the nineteenth century, when sugar became widely available at a low cost due to colonial trade. Of interest, the consumption of sugar was tightly linked with that of sweetened beverages, initially tea, coffee, and chocolate in the nineteenth century and later sodas at the beginning of the twentieth century [1, 2].

The debate about the effects of sugar on health is far from recent. In 1912, the French physician Paul Carton proposed [3] that human diseases were mainly related to the consumption of three *deadly nutrients*: alcohol, meat, and industrial sugar. In the 1960s, Yudkin proposed that *pure*, *white*, and *deadly* sugar, more than saturated fat, was instrumental in causing cardiovascular diseases [4, 5]. The adverse effects of sugars remained however controversial, and another French physician, Gerard Debry, concluded after an extensive review of the literature until 1995 that there was little evidence to support a harmful effect of sugars [6].

The debate about sugar was relaunched in 2004, when Bray, Niels, and Popkins suspected a causal relationship between the increased consumption of high-fructose corn syrup (HFCS) and the rise in the obesity prevalence in the USA [7]. They proposed that fructose was specifically responsible for adverse health effects due to its specific metabolism. The initial *whistle blowing* incriminating novel industrially produced sweeteners was, in part, linked with the incorrect assumption that HFCS contained more fructose than sucrose. The actual fructose content of HFCS is somewhere between 42 and 55 % total sugars and therefore shows little difference with sucrose. Several clinical studies comparing the effects of HFCS and sucrose concluded that these two sweeteners have very similar effects [8–10]. Nonetheless, many strong position statements were published in the scientific literature and in the lay press stating that sugar, and more specifically its fructose component, is a *toxic substance* and a major determinant of non-communicable diseases [11–14].

Since this initial renewed interest in fructose-containing caloric sweeteners (FCCS), many research studies have been performed and many articles have been published, but the controversy continues. Several health organizations however concluded that consumption of added sugar should be drastically reduced to less than 5 % total energy (http://www.who.int/nutrition/sugars_public_consultation/en/, https://www.gov.uk/government/consultations/consultation-on-draft-sacn-carbohydrates-and-health-report); these recommendations appear at odds with reports from European Food Safety Agency [15] and the Institute of Medicine [16] which both concluded that scientific evidence was lacking to set up an upper level for sugar consumption.

In this position paper, we attempt to define what we have learned during these past 10 years, which questions have been unequivocally answered, and which novel questions arose. In this process, we did not perform a critical, systematic review of the impressive literature on the topic but mainly focused on relevant meta-analyses and critical, systematic reviews published between 2007 and February 2015.

## Why Did We Initially Suspect That FCCS Are Deleterious to Metabolic Health?

A role of FCCS in the pathogenesis of metabolic diseases was originally supported by

- Animal studies, mainly performed in rodents, but also in other animal species, including non-human primates, which indicate that adding fructose or sucrose to the diet can lead to the development of obesity, insulin resistance, type 2 diabetes, dyslipidemia, and occasionally high blood pressure [17, 18]. In most of these studies, high-sugar-fed animals are used as an experimental model of obesity or metabolic diseases, and the effects of a high FCCS intake are not directly compared to other obesogenic high-fat-based diets which produce similar long-term metabolic alterations [19, 20]. As such, they unequivocally demonstrate that sugar can induce overeating, obesity, and metabolic diseases in spontaneously feeding animals, but come short of demonstrating that this effect is exclusively related to FCCS.
- A small number of human studies showing that a high-fructose diet was occasionally associated with the development of dyslipidemia [21].
- Epidemiological data showing associations between FCCS intake and body weight or prevalence/incidence of metabolic diseases [7, 22–27].
- Allegations that fructose may be obesogenic because of its propension to stimulate de novo lipogenesis [23–27] or may elicit inadequate suppression of food intake because it does not increase the secretion of insulin and gut satietogenic hormones [28, 29].

## What Has Been Learned Over the Past 10 Years?

Far too many original articles and reviews were published in the scientific literature during this period to review each one in detail here. Evaluating the impact of this large amount of studies can however be facilitated by several recent meta-analyses and critical, systematic reviews that can be grossly classified into (a) epidemiological studies, (b) clinical intervention studies, and (c) controlled mechanistic studies. The major meta-analyses published between 2007 and February 2015 [30–38, 39•, 40–42, 43••, 44, 45, 46••, 47, 48••, 49••, 50–52] are listed in Table 1, together with the type of studies included in the meta-analysis, its major results, and their authors’ main conclusions.

**Table 1:** Selected meta-analysis and systematic reviews having addressed the effects of fructose-containing caloric sweeteners on human health

Publication	Review focus	Number of studies	Main results	Authors’ conclusions
Kelishadi, R et al., Nutrition 2014 [32]	Association between fructose consumption and components of the metabolic syndrome	15 controlled mechanistic studies	Fructose consumption positively associated with fasting blood sugar, fasting blood triglycerides, and systolic blood pressure and negatively associated with HDL cholesterol	Fructose consumption from industrialized food has significant effects on most components of the metabolic syndrome
Wang et al., Atherosclerosis 2014 [34]	Effect of fructose on postprandial triglycerides	14 mechanistic studies	Increased postprandial triglyceride concentration when fructose administered together with an excess caloric intake	Fructose in isocaloric exchange for other carbohydrate does not raise postprandial triglycerides. A small effect, however, cannot be ruled out under all isocaloric conditions. In contrast, there is a consistent and substantial postprandial triglyceride-raising effect of fructose seen in hypercaloric trials, in which fructose supplements background diets with excess energy at extreme doses
Te Morenga, L. et al., Am. J. Clin. Nutr. 2014 [49••]	Association between dietary sugars and lipids/blood pressure in adults and children	40 mechanistic or intervention studies	Plasma triglyceride, total, and LDL cholesterol were higher, and HDL cholesterol was lower in individuals with higher sugar intake. The relationship between sugar intake and lipids was most marked when sugar was administered in weight-maintenance diet and body weight did not change. Sugar intake was associated with higher systolic and diastolic blood pressure	Dietary sugars influence blood lipids and blood pressure independent of effects on body weight
Cheungpasitporn, W et al., Nephrology 2014 [39•]	Association of sugar-sweetened beverages and chronic kidney disease	2 prospective cohort studies and 3 cross-sectional or case-control studies	Relative risk of chronic kidney disease was significantly higher in subjects consuming sugar-sweetened beverages	This finding suggests that sugar-sweetened beverages may impact clinical management and primary prevention in high-risk patients
Jayalath, VH et al., J. Am. Coll. Nutr. 2014 [33]	Association between total fructose intake and risk of hypertension	3 prospective cohort studies	No association between fructose intake and risk of hypertension	Total fructose intake is not associated with increased risk of hypertension
Chung, M et al., Am. J. Clin. Nutr 2014 [35]	Effects of fructose-containing caloric sweeteners and non-alcoholic fatty liver diseases and markers of liver health	21 controlled mechanistic studies	Low levels of evidence that hypercaloric fructose increases intrahepatic fat content and plasma AST concentrations	The apparent association between liver health and fructose-containing caloric sweetener intake appears to be confounded by excessive energy intake
Xi, B et al., PLoS ONE 2014 [30]	Association between intake of fruit juices and incidence of type 2 diabetes	4 prospective cohort studies	Relative risk of type 2 diabetes was significantly increased with consumption of sugar-sweetened fruit juices, but not with consumption of 100 % fruit juice	Our findings support dietary recommendations to limit sugar-sweetened beverages such as fruit juice with added sugar
Chiu S et al., Eur. J. Clin. Nutr. 2014 [36]	Effects of fructose on markers of non-alcoholic fatty liver diseases	13 controlled mechanistic studies	Fructose in weight-maintenance diet had no effects; fructose in hypercaloric diet increased intrahepatic lipid concentration and blood ALT	Fructose providing excess energy at extreme doses raises intrahepatic fat and ALT, an effect that may be attributable to excess energy intake
Greenwood DC et al., Br. J. Nutr 2014 [51]	Association between sugar-sweetened and artificially sweetened soft drinks and type 2 diabetes in prospective cohorts	11 epidemiological prospective studies	Relative risk increases by 120/330 ml/day with sugar- and by 113/330 ml/day with artificially sweetened drinks; the relationship is attenuated when data was adjusted for BMI	Findings indicate a positive association between sugar-sweetened soft drink intake and type 2 diabetes, attenuated by adjustment for BMI. The same association is observed with artificially sweetened beverages. Possible reverse causality
Malik, VS et al., Am. J. Clin. Nutr 2013 [31]	Effects of sugar-sweetened beverages on weight gain in adults and children	32 (7 epidemiological prospective studies and 5 clinical intervention studies in children, 7 epidemiological prospective studies and 5 clinical intervention studies in adults)	In cohort studies, every daily serving of sugar-sweetened beverage is associated with body weight gain in children (+0.06 unit increase in BMI/year in children; +0.22 kg/year in adults). RCTs in children showed decreased body weight with reduced sugar-sweetened beverage consumption; RCTs in adults showed increased body weight with increased sugar-sweetened beverage consumption	Sugar-sweetened beverage consumption promotes weight gain in children and adults
Zhang, YH et al., J. Nutr 2013 [37]	Effects of very-high-fructose intake on total and LDL cholesterol	24 mechanistic studies	Fructose dose dependently increases total and LDL cholesterol for daily intake >100 g; no effect for daily fructose intake <100 g. No effect on HDL cholesterol at any level of intake	Very-high-fructose intake (>100 g/day) increases total and LDL cholesterol
Pan A et al., Int. J. Obesity 2013 [40]	Effects of changes in water and sweetened beverage intake on long-term weight changes	3 prospective cohort studies	Participants gained on average 1.5 kg/4 years; 1 cup/day increment in sugar-sweetened beverage associated with 0.36 kg body weight gain/4 years, 1 cup/day fruit juice with 0.22 kg/4 years body weight gain	Results suggest that increasing water intake in place of sugar-sweetened beverages is associated with lower long-term weight gain
Te Morenga, L et al., BMJ 2013 [48••]	Association between dietary sugars and body weight in adults and children	38 prospective cohort studies and 30 mechanistic or intervention studies	In adults, increased sugar intake was associated with increased body weight and isoenergetic exchange of sugar with other carbohydrate with no change in body weight; in children, recommendation to decrease added sugar intake did not result in body weight changes, but in prospective cohort studies, the odds ratio for being overweight was significantly higher in the group with the highest intake of sugar-sweetened beverages	Among free-living people involving ad libitum diets, intake of added sugars or sugar-sweetened beverages is a determinant of body weight. Changes which occur with modifying intakes seem to be mediated via changes in energy intake
Cozma AI et al., Diab. Care 2012 [46••]	Effects of fructose on glycemic control in diabetes	18 controlled mechanistic studies	Isocaloric exchange of fructose for other carbohydrates reduced glycated blood proteins	Isocaloric substitution of fructose for other carbohydrates improves long-term glycemic control in diabetes
Ha, V et al., Hypertension 2012 [45]	Effects of fructose on blood pressure	Mechanistic studies	Fructose intake in 13 trials with weight-maintenance diets decreased diastolic and mean blood pressure and had no effect on systolic blood pressure; fructose intake in 2 RCTs with hypercaloric diet had no effect on blood pressure	No adverse effect of isocaloric substitution of fructose for other carbohydrates on blood pressure
Sievenpiper, JL et al., Ann Int. Med. 2012 [43••]	Effects of fructose on body weight	41 mechanistic and intervention studies	Fructose included in a weight-maintenance diet has no effect on body weight; fructose included in an hypercaloric diet significantly increases body weight	Fructose does not seem to cause weight gain when substituted for other carbohydrates in diets providing similar amounts of calories
Mattes, RD et al., Obesity Rev 2011 [41]	Effects of changes in sugar-sweetened beverage consumption on body weight	12 intervention studies	6 RCTs that added sugar-sweetened beverages showed dose-dependant increases in body weight; 6 RCTs that decreased sugar-sweetened beverages showed no effect on body mass index	The current evidence does not demonstrate conclusively that reducing sugar-sweetened beverages will reduce BMI levels
Malik, VS et al., Diab. Care 2010 [50]	Association between sugar-sweetened beverage intake and risk of metabolic syndrome and diabetes in prospective cohort studies	11 epidemiological prospective studies	In 8 prospective studies, the participants in the highest quantile for sugar-sweetened beverage consumption (1–2 servings/day) had a 28 % increased risk of developing diabetes. In 3 cohort studies, relative risk for developing the metabolic syndrome was increased by 20 % in high consumers	In addition to weight gain, higher consumption of sugar-sweetened beverages is associated with the development of metabolic syndrome and type 2 diabetes
Sievenpiper JL et al., Diab. Care 2009 [47]	Effects of fructose on blood lipids in subjects with type 2 diabetes	16 controlled mechanistic studies	Fructose replacing starch increased blood triglyceride when dose >60 g/day or follow-up was <4 weeks	Threshold dose for fructose increasing blood triglyceride in subjects with type 2 diabetes is 60 g/day
Forshee, RA et al., Am. J. Clin. Nutr 2009 [52]	Association between sugar-sweetened beverage intake and body mass index in children and adolescents	8 epidemiologic prospective studies and 2 clinical intervention trials	No significant association between sugar-sweetened beverage consumption and BMI	The association between sugar-sweetened beverages consumption and body mass index is zero based on scientific evidence as of 2009
Livesey, G et al., Am J Clin Nutr 2008 [21]	Effects of fructose on glycated hemoglobin (HbA1c) and blood triglycerides	42 mechanistic and intervention studies	Lower HbA1c with fructose intake <90 g/day; no significant effects on fasting blood triglycerides for fructose intake <100 g/day; no effect on postprandial blood triglyceride for intake <50 g/day	Fructose intake from 0 to 90 g/day has a beneficial effect on HbA1c; significant effects on blood triglycerides are not observed unless fructose intake >50 g/day

*Epidemiological studies* are based on analysis of prospective data available from large cohort of subjects with multiple dietary evaluations over time and several years of follow-up on body weight or incidence of metabolic diseases. Such studies allow identifying statistical associations between dietary intake at initial assessment or dietary changes observed between two dietary assessments and subsequent health status. As such, they are highly valuable to assess the plausibility that FCCS may be associated with metabolic diseases in *real-life* situations [53]. They however do not allow identifying causal relationship between FCCS and health due to the many possible confounders [54].

Epidemiological prospective studies show a strong association between FCCS intake and body weight gain [40, 55, 56••]. Most studies specifically addressed the effects of sugar-sweetened beverage (SSB) consumption, however, and the association between consumption of sugar in solid foods and obesity is relatively understudied. The results indicate that SSB consumption is strongly associated with higher total energy intakes [57], and that either SSB, total fructose, or total FCCS consumption is strongly associated with body weight gain over time. They however also indicate that other dietary food components, mainly fried potatoes, red meat, and processed meat, are also involved in body weight gain [56••]. Furthermore, FCCS consumption is also associated with the incidence of dyslipidemia, insulin resistance, and type 2 diabetes [58], with incidence or risk factors for cardiovascular diseases [59–62], with cardiovascular mortality [63•], with chronic kidney diseases [39•], and with hyperuricemia and gout [64]. The strength of these associations is generally reduced when data are adjusted for body weight, suggesting that they are, at least in part, secondary to increased body fat mass.

What can be concluded at this stage? The epidemiological data available so far are quite consistent in pointing toward a positive association between FCCS consumption and the development of obesity. The association is particularly robust for SSB. These studies further indicate that the association between SSB consumption and body weight is largely due to SSB being associated with increased total energy intake [31, 48••].

*Clinical intervention studies* on FCCS regroup various clinical trials aimed at assessing the global effects of changes in FCCS consumption on pre-defined endpoints in specific subsets of the population. The intervention here is focused on FCCS consumption alone (e.g., replacing SSB with water or artificially sweetened beverages, or adding one or several servings of SSB per day), while otherwise leaving spontaneous food and beverage intake and physical activity uncontrolled. Intervention clinical trials answer one specific question, that is: will a specified intervention (e.g., reduction of sugar intake or of SSB intake) be effective in reaching a specified endpoint (e.g., body weight loss or decreased blood triglyceride concentrations)? As such, they are valuable to evaluate whether it may be worth to introduce the intervention into clinical practice or into public health initiatives. Identification of underlying mechanism may however be limited because more than one mechanism may account for variations of the main outcome. For instance, an intervention trial showing a beneficial effect of reducing FCCS intake may indicate that FCCS is specifically obesogenic; the same result may however also be explained to a reduction in total energy intake (which suggests that reduction of energy intake from other nutrients may have had the same effect). Conversely, if the same intervention trial fails to observe beneficial effects of FCCS reduction, this may reflect that FCCS do not exert specific adverse effects; alternatively, this may also be explained by the absence of compliance (i.e., the participants failing to comply with the dietary instructions) or by a compensation of FCCS energy by an increased consumption of other foods.

Many clinical intervention studies have assessed the effects of reducing or increasing SSB intake in adults and children. These studies have been further analyzed in very complete meta-analyses [31, 48••]. Increased consumption of SSB has been shown to result in significant weight gain compared to control in both adults and children, and this effect has been attributed essentially to an increased total energy intake. Changes in body weight observed in some of these studies were however much lower than what would have been expected from cumulated total energy from SSB [65••, 66••, 67, 68]. Reduction of SSB consumption was generally associated with significant weight loss and reduced total energy intake. Here again, in many trials, weight loss was far less than what would have been expected based on cumulated SSB energy deficit, suggesting either low compliance to SSB restriction or compensatory increase in total energy intake. Reducing SSB intake was however inconsistently efficient in children, most likely due to low compliance in this class of age [31, 48••].

What have we learned from intervention trials? First, many trials have assessed the effects of adding sugar to the diet. Quite expectedly, increasing sugar consumption was associated with some weight gain. The effects of increasing sugar consumption was not compared to those of increasing starch or fat consumption, and hence, these studies fall short of demonstrating that weight gain is specifically related to sugar. Second, most studies showed a significant reduction of body weight when SSB consumption was reduced, which certainly indicates that the intervention significantly decreased total energy intake. Although the body weights reported in the intervention and control arms were statistically significant, the actual effect on adiposity was often very low: in one study, BMI increased from 30.4 to 30.5 kg/m^2^ in obese adolescent who replaced SSBs with water and from 30.1 to 30.6 kg/m^2^ in the control group [66••]; in another study, body weight non-significantly increased by 1.3 % in adolescent receiving SSBs for 6 months, compared to 0.2 and 0.6 % in those receiving artificially sweetened sodas or water, respectively [69•]. This casts serious doubt on the clinical efficacy of a reduction of SSB consumption alone.

*Controlled mechanistic studies* consist of short-term intervention studies in selected groups of individuals, most often healthy subjects, overweight and obese subjects, or subjects with type 2 diabetes. The experimental protocols compare an intervention arm with high FCCS diet to control arms, which can be either a low FCCS weight-maintenance diet or isocaloric high-glucose/high-starch/high-fat diets. These studies specifically address what might be the consequences of a high FCCS and attempt to identify potential mechanisms underlying the effects of excess FCCS. Given their strictly controlled conditions, each mechanistic study aims at answering a specific question (e.g., what are the effects of isocaloric amounts of sucrose vs. HFCS on blood lipids? Is fructose more lipogenic than isocaloric amount of glucose? Can excess fructose impair insulin sensitivity?). Results from mechanistic studies merely assess whether a specific role of FCCS is plausible as a working hypothesis. They however cannot assess whether such effects are relevant to the general population in free-living conditions.

Total energy intake is an important confounding factor in these studies. Studies having compared excess energy intake from FCCS to weight-maintenance, low-FCCS diets consistently demonstrate that excess FCCS energy consumption can, within a few days to a few weeks, increase body weight and body fat stores [70], enhance hepatic glucose production [71], impair hepatic suppression of glucose production by insulin [71–73], increase fasting and postprandial blood triglyceride concentration [29, 70, 74, 75], increase intrahepatic fat concentration [76•], and cause a rise in blood uric acid concentrations [70, 77]. Two studies suggested that excess fructose may increase visceral fat mass while excess glucose would increase mainly subcutaneous fat [70]. In one of these studies [70], fructose-induced increase in visceral fat was observed only in males, but not in females. Altogether, these mechanistic studies unfortunately cannot sort out the relative roles of FCCS and energy intakes. In addition, many of these studies assessed the effects of pure fructose rather than sucrose or HFCS, and many of them used levels of daily FCCS intake well above consumption observed in the general population.

Hypercaloric FCCS diets consistently caused an increase in blood triglyceride and uric acid concentrations, while hypercaloric high-glucose or high-fat diets did not. One meta-analysis concluded that high-fructose intake was associated with an increased blood triglyceride only when associated with excess energy intake, but had no effects when part of a weight-maintenance diet [34]. In contrast, another study concluded that dietary sugars influence blood lipids independently of body weight [49••]. One meta-analysis specifically addressed the effects of sugar on blood cholesterol and reported that adverse effects of sugars were observed for doses higher than 100 g/day [37].

Many narrative reviews and position papers propose that insulin resistance is a well-recognized effect of dietary fructose [78–80]. Moderate increases in fasting hepatic glucose production are indeed observed within a few days on a high-fructose diet but are not associated with clinically relevant increases in blood glucose. Surprisingly, all studies having actually assessed muscle insulin sensitivity by hyperinsulinemic-euglycemic clamps failed to report any muscle insulin resistance in healthy individuals after 1–4 weeks on a high-fructose diet [71–73, 81] or in subjects with type 2 diabetes after 3 months [82]. This strongly suggests that fructose per se, independently of long-term weight changes, does not impair insulin’s actions in muscle. Interestingly, one study observed a significant muscle insulin resistance in middle-aged, overweight offspring of type 2 diabetes subjects after 6 days on a high-fructose diet [83], while another similar study, performed in young, non-overweight offspring of type 2 diabetes, failed to document any changes [81]. This raises the possibility that fructose’s effects may be modulated by both genetic background and additional factors such as age or body fat mass.

Whether FCCS are responsible for an excess food intake through specific effects involving food intake control has been the focus of active research and will not be reviewed comprehensively here. It has been proposed that fructose fails to inhibit the release of the orexigenic gut hormone ghrelin and increases the release of satiating gut hormones such as GLP-1 and PYY to a lesser extent than glucose or starch [29]. This concept has however been challenged by a recent study showing that fructose had substantial effects on these gut hormones in normal-weight adolescent. The same study however reported an impaired suppression of acyl-ghrelin, the active form of ghrelin, in obese, insulin-resistant adolescent [84]. Furthermore, the hypothesis that fructose may exert less suppression of food intake than glucose or complex carbohydrate is based on indirect markers of food intake control, which relates essentially to the so-called homeostatic systems of food intake regulation, located in the brain stem and hypothalamus. Studies with direct measures of food intake and/or satiety feeling failed to document any difference between fructose and other caloric sweeteners in humans [28, 85, 86]. Food intake regulation results from complex interactions between metabolic, hormonal, and neurologic signals [87]. There is growing evidence that the hedonic system of food intake, located in neocortical structures, and which is involved in food preferences and palatability, plays a prominent role in food intake control [88]. It has further been suggested, based on the fact that oral sugar ingestion stimulates dopaminergic neurons of the *reward system* of the brain stem, hypothalamus, and cortex, that this may confer to FCCS potentially addictive potential, at least in animals [89–92] and possibly in humans as well [93]. These are early hypotheses, which still need more studies to be fully evaluated. The concept of sugar addiction is highly debated but suffers from lack of clear diagnosis criteria. Finally, there is to date no scientific evidence that in humans, sugars stimulate these neurological pathways to a larger extent than other food substances with similar palatable properties.

The effect of intravenous fructose administration on uric acid production and to cause hyperuricemia has been long known [94]. Interesting novel observations have recently been made to link FCCS-induced uric acid production to metabolic and cardiovascular diseases. According to these novel hypotheses, uric acid may act as a mediator to induce endothelial cell dysfunction, thus preventing insulin-induced muscle vasodilation. This effect of uric acid has been postulated to contribute to fructose-induced insulin resistance and high blood pressure [95]. One isolated clinical study has supported this hypothesis by showing that a short-term hypercaloric high-fructose diet increased blood pressure and that this effect was prevented by administration of the uric acid synthesis inhibitor allopurinol [77]. This observation must however be tempered by the fact that several other studies failed to observe a significant effect of a high-fructose diet on blood pressure [45]. Besides vascular effects, uric acid has also been proposed to enhance lipogenesis and thus to contribute to dyslipidemia and hepatic steatosis [96, 97, 98••]. These novel hypotheses clearly require to be further evaluated, and more human studies are needed to assess their clinical relevance.

What clinically relevant information do these mechanistic studies offer? There is robust evidence that a high-fructose diet can increase blood triglycerides (TG) within a few days, enhance IHCL storage, and increase endogenous glucose production. These changes are highly significant, yet of small magnitudes, and blood triglyceride and glucose concentration, as well as intrahepatic fat content, remain generally within the normal range. They may of course be early markers of insulin resistance and progress with time to the development of metabolic diseases. There is however an alternative explanation which has been widely disregarded until now, i.e., that these changes may reflect mere adaptation to a high-fructose diet.

**Fig. 1:**
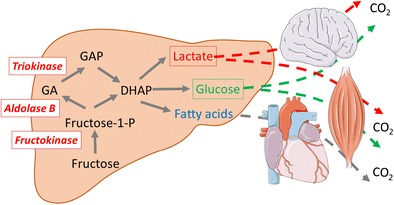
Unlike glucose, which can be used as an energy substrate by all human cells, fructose cannot be directly metabolized by most cells due to a much lower affinity of hexokinases for fructose than for glucose. Instead, fructose is first metabolized in a limited number of organs (liver, small intestinal mucosa, kidney). In these organs, it is metabolized to trioses-phosphate (di-hydroxyacetone-phosphate and glyceraldehyde-3-phosphate) by a set of specific enzymes: fructokinase (ketohexokinase), aldolase B, and triokinase. Trioses-phosphate can subsequently be further converted into ubiquitous energy substrates: lactate, glucose, and fatty acids (palmitate, oleate, stearate, …)

**Fig. 2:**
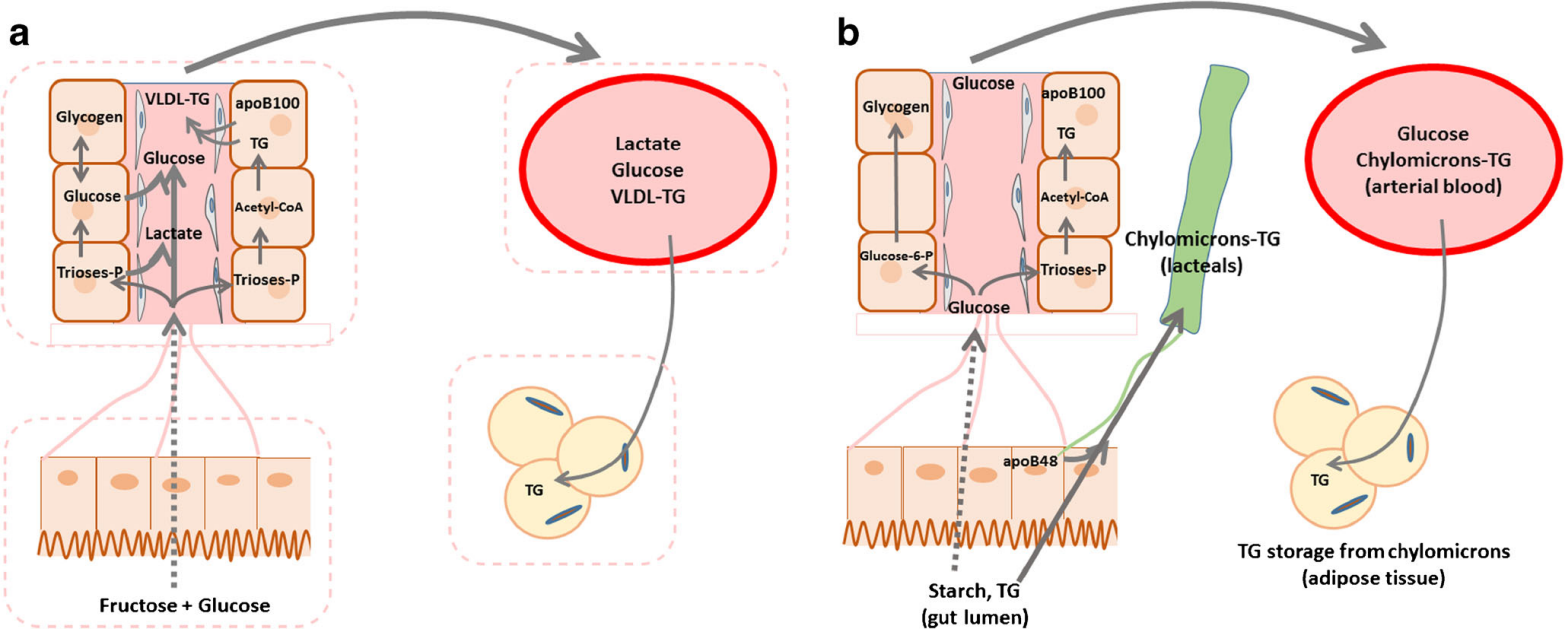
Pathways used for lipid storage from dietary fructose and dietary fat. Fructose (**a**) is essentially extracted and metabolized in the liver, where part of it can be converted into lactate, glucose, fatty acids, and triglycerides. Newly synthesized glycogen can be temporarily stored as hepatic glycogen, but its major portion is slowly released into the systemic circulation as glucose. Hepatic fructose handling differs from glucose (whether ingested as free glucose, sucrose, or starch; **b**), which is only partially extracted by the liver to replenish glycogen stores. Its largest portion however reaches the systemic circulation, elicits an increase in blood glucose and insulin concentration, and is metabolized in insulin-sensitive tissues (muscle, adipose tissue) within a few hours. After fructose ingestion, new fat synthesized de novo in the liver can be temporarily stored within the hepatocytes and/or be secreted with VLDL to be secondarily deposited in adipose tissue (**a**). In contrast, ingested fat is mainly absorbed as chylomicrons which circulates in the lymph, thus bypassing the liver; chylomicrons-TG will then join the systemic circulation and be deposited in adipose tissue (**b**)

Unlike fructose, glucose and fatty acids are key energy substrates for animals, and most cells of the human organism synthesize the enzymes required for glycolysis, beta-oxidation, and degradation of glucose- or fat-derived acetyl-CoA in the Krebs cycle, coupled to ATP synthesis in the respiratory chain. In contrast, fructose cannot be used as such by most cells and requires to be first pre-processed into glucose, lactate, or fatty acids [99]. Specific fructose-metabolizing enzymes, fructokinase, aldolase B, and triokinase, are present mainly not only in the liver but also in enterocytes and tubular kidney cells where this pre-processing occurs (Fig. 1). In the liver, fructolysis, unlike glycolysis, is neither regulated by insulin nor inhibited by high concentrations of ATP or citrate. Hepatic production of glucose, lactate, and fatty acids is therefore mainly dependant on the amount of fructose ingested with a meal or a drink. The first adaptation that may occur as a result of fructose ingestion is an increase in hepatic glucose production [71–73, 100]. As fructose is initially converted to lactate and glucose in the liver, the resulting increased blood lactate and the modest increase in hepatic glucose production observed in healthy subjects consuming a high-fructose diet do not come as a real surprise. The second adaption consists in the well-characterized increase in plasma lipids. However, the metabolic pathways involved markedly differ from those resulting from dietary fat ingestion: fructose conversion into fat occurs essentially in the liver where fructose-derived fat is either temporarily stored as intrahepatic fat and/or secreted into the blood as VLDL triglyceride. In contrast, dietary fats absorbed from the gut are released as chylomicrons in the lymph, thus avoiding first-pass hepatic metabolism to be directly stored in peripheral adipocytes. This difference in the interorgan trafficking involved in the handling and storage of these two lipid sources most likely accounts for increased blood triglyceride concentrations in high-fructose-fed subjects, since chylomicrons-TG have a half-life in circulation considerably faster than VLDL (Fig. 2).

Whether these alterations of glucose and lipid metabolism following fructose ingestion are mere adaptations to a transient change in diet or will in the long term be causally associated with adverse vascular effects remains unknown. If that was the case, it would be important to gather further clinical observation in order to determine whether such deleterious effects of FCCS are restricted to the subgroup of subjects at increased risk due to their genetic background or to specific patterns of FCCS consumption.

## Conclusions

Scientific data regarding the effects of fructose-containing caloric sweeteners presently indicate clearly that

1. Sugar consumption has reached record values in man’s history during the second half of the twentieth century and the beginning of the twenty-first century and represents on average close to 20 % total energy intake at the population level in developed countries.
2. Sugar consumption is consistently associated with increased energy intake and with the development of obesity and metabolic diseases in epidemiological studies.
3. Fructose can cause weight gain when present in a high-energy diet providing more calories than required for weight maintenance. There is however no evidence that similar effects would not be observed when replacing sugar with glucose, starch, or lipids.
4. A high-fructose diet can increase blood triglyceride, alter hepatic glucose output, and increase uric acid concentrations. Whether these effects would increase the risk of metabolic or cardiovascular disease independently of an increase in body fat mass remains speculative.

## Between Science and Common Sense: Which Practical Steps May Be Considered?

The scientific data reported in the literature appears somewhat conflicting at first sight. On one hand, there is no strong evidence that, at similar intake levels, sugar exerts worse deleterious effects than other energy substrates. Whether sugar would specifically impair food intake control, or cause tissue lipotoxicity, remains open questions, but levels of evidence are presently too low to lead to specific recommendations. On the other hand, a huge proportion of the population of an ever-increasing number of countries is overweight. As a nutrient accounting to 15–20 % total energy, sugar is certainly contributing to this global excess energy intake [101–103]. Furthermore, many sugary products have a low content of essential nutrients and hence low *nutritional quality*, which makes them a potential target for energy reduction. This is particularly true of SSBs. There is no strong evidence that energy consumed with beverages is more obesogenic than energy consumed with solid foods and no plausible mechanism to account for such an effect. SSB may nonetheless be easily overconsumed, possibly because beverage intake is stimulated by thirst stimuli, such as blood hyperosmolarity and sodium levels in the distal renal tubules, irrespective of energy balance. As such, SSB reduction is certainly a prime target for the prevention of metabolic disorders, assuming that such decrease will not be compensated by the increase in caloric intake from solid food.

However, if sugars contribute to excess energy intake, there is also compelling evidence that sugar-devoid foods, such as potato chips and meat, are also involved [56••]. Furthermore, interventions focused solely on SSB or sugar intake have failed to achieve clinically relevant weight reduction. The excess body weight of the US population corresponds to a ca. 350–500 kcal/day excess energy intake on average [104]. In order to revert obesity, energy intake should be reduced by the same amount; in order to reach this target by specifically decreasing sugar intake, added sugar consumption should be reduced to close to zero, which may be unrealistic in the near future. Reduction of SSBs, and reformulation of sugar-rich industrial foods, may certainly contribute to reduced total sugar and energy intake, but it appears obvious that the consumption of other energy-dense foods and/or multifactorial interventions including consumer education, diet, and physical activity will be needed to achieve these goals.
